# Melatonin Analogue Antiproliferative and Cytotoxic Effects on Human Prostate Cancer Cells

**DOI:** 10.3390/ijms19051505

**Published:** 2018-05-18

**Authors:** Angela Calastretti, Giuliana Gatti, Valeria Lucini, Silvana Dugnani, Gianfranco Canti, Francesco Scaglione, Annamaria Bevilacqua

**Affiliations:** 1 Department of Medical Biotechnology and Translational Medicine, Università degli Studi di Milano, 20122 Milan, Italy; luisa.calastretti@unimi.it (A.C.); giuliana.gatti@unimi.it (G.G.); gianfranco.canti@unimi.it (G.C.); 2 Department of Oncology and Hemato-oncology, Università degli Studi di Milano, 20122 Milan, Italy; valeria.lucini@unimi.it (V.L.); silvana.dugnani@unimi.it (S.D.); francesco.scaglione@unimi.it (F.S.)

**Keywords:** melatonin analogues, melatonin receptors, anti-cancer drugs, prostate cancer, androgen receptor, Akt

## Abstract

Melatonin has been indicated as a possible oncostatic agent in different types of cancer, its antiproliferative role being demonstrated in several in vitro and in vivo experimental models of tumors. Specifically, melatonin was proven to inhibit cell growth of both androgen-dependent and independent prostate cancer cells, through various mechanisms. A number of melatonin derivatives have been developed and tested for their role in the prevention and treatment of neoplastic diseases. We recently proved the in vitro and in vivo anticancer activity of UCM 1037, a newly-synthetized melatonin analogue, on melanoma and breast cancer cells. In this study we evaluated UCM 1037 effects on cell proliferation, cell cycle distribution, and cytotoxicity in LNCaP, PC3, DU145, and 22Rv1 prostate cancer cells. We demonstrated significant dose- and time-dependent UCM 1037 antiproliferative effects in androgen-sensitive LNCaP and 22Rv1 cells. Data from flow cytometric studies suggest that UCM 1037 is highly cytotoxic in androgen-sensitive prostate cancer cells, although no substantial increase in the apoptotic cell fraction has been observed. UCM 1037 cytotoxic effects were much less evident in androgen-insensitive PC3 and DU145 cells. Experiments performed to gain insights into the possible mechanism of action of the melatonin derivative revealed that UCM 1037 down-regulates androgen receptor levels and Akt activation in LNCaP and 22Rv1 cells.

## 1. Introduction

Melatonin is an indolic hormone which plays pleiotropic roles and is widely distributed in most living organisms, where it is involved in various physiological functions [1]. In mammals, melatonin is recognized as the key regulator of the circadian rhythm and is largely secreted by the pineal gland in response to darkness [2]. However, many other organs or cells producing melatonin have been identified, including the gastrointestinal tract, skin, reproductive tract, and immune system cells [3]. The relevance of melatonin has been demonstrated in human physiology and pathology, due to its anti-inflammatory properties, antioxidant action, and its role in immunomodulation, energy metabolism, and hematopoiesis.

Furthermore, a number of epidemiological studies support a protective role of melatonin in both hormone-dependent and hormone-independent cancers, suggesting an inverse correlation between melatonin level and cancer incidence [4]. Specifically, two case-control studies were conducted on prostate cancer patients indicating that men with urinary melatonin levels below the median had a statistically significant increased risk to develop advanced disease compared with men with levels above the median [5,6].

Melatonin has oncostatic properties in different in vitro and in vivo experimental models of neoplasia [7]. The effects of melatonin are mediated by both receptor-dependent and receptor-independent mechanisms. Melatonin receptors include plasma membrane and nuclear binding sites. Two membrane receptors, MT_1_ and MT_2_, belonging to the family of guanine nucleotide-binding regulatory protein (G protein)-coupled receptors, have been characterized in mammals [8]. MT_1_ activation leads to diverse responses, most of them acting on cAMP signaling and the calcium-calmodulin pathway, although the signal transduction pathways response varies among different tissues and cell types [9]. Binding of melatonin to MT_2_ receptors triggers a number of signal transduction pathways including phosphoinositide production, the inhibition of adenylyl cyclase and the inhibition of soluble guanylyl cyclase pathway [10]. Both membrane receptors can also activate other signaling pathways involved in multiple regulatory processes, including phospholipase C/protein kinase C, mitogen-activated protein kinases, and extracellular-signal-regulated kinase pathways [3,11]. Melatonin receptors have been detected in numerous tissues and are widely distributed in the body [12].

MT_1_ and MT_2_ expression was also shown in human prostate cancer cells [13,14]; however, there is still controversy regarding the exact role of these receptors in melatonin-mediated protection against prostate cancer progression. Melatonin receptor-independent mechanisms have been demonstrated in prostate cancer suggesting a scenario where the indole triggers signaling cascades that ultimately hamper prostate cancer cell growth [7].

Several melatonin derivatives have been recently developed and tested for their role in the prevention or treatment of neoplastic diseases, either alone or in combination with other drugs to improve the sensitivity of cancers to inhibition by conventional drugs, and to reduce side-effects [15,16]. We previously characterized a number of melatonin receptor ligands and we demonstrated the in vitro and in vivo anticancer activity of newly-synthetized melatonin analogues on melanoma and breast cancer cells [17]. In particular, UCM 1037 melatonin derivative demonstrated significant antiproliferative effect in DX3, WM-115, MCF-7, and MDA-MB231 cells; the flow cytometric analysis also confirmed the cytotoxic activity of UCM 1037 on the same cell lines. The anti-tumor activity of UCM 1037 was also demonstrated in vivo in a melanoma xenograft mice model.

In this paper, UCM 1037 melatonin analogue effects were evaluated in different prostate cancer cell lines. We examined the effects of the molecule on cell proliferation, cell cycle distribution, and cytotoxicity. Experiments were also performed to gain insights into the possible mechanism of action of the melatonin derivative and its crosstalk with the androgen signaling pathway.

## 2. Results

### 2.1. Expression of Melatonin Receptors in Prostate Cancer Cells

**Figure 1:**
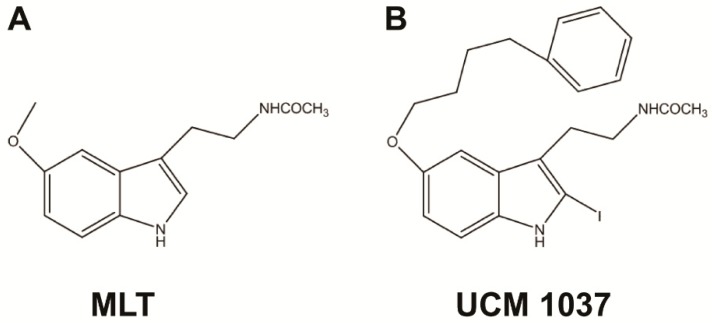
Chemical structures of melatonin (MLT) (**A**) and its synthetic derivative UCM 1037 (**B**).

In order to evaluate the antiproliferative role of the previously characterized melatonin analogue UCM 1037 (Figure 1) on human prostate cancer cells, four cell lines were selected.

**Figure 2:**
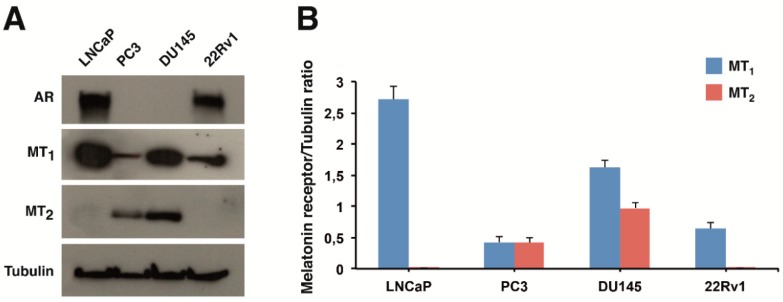
AR, MT_1_, and MT_2_ protein levels in prostate cancer cell lines. (**A**) Western blot showing the expression of AR, MT_1_, and MT_2_ in LNCaP, PC3, DU145, and 22Rv1 cells. Tubulin is shown as a loading control. One representative blot is shown of three independent experiments; (**B**) densitometry of MT_1_ and MT_2_ protein expression. Bar charts show quantification of MT_1_ (blue bars) and MT_2_ (red bars) normalized versus tubulin in prostate cancer cell lines. Each bar represents the mean ± standard deviation (s.d.) of three independent experiments.

We chose both androgen-sensitive, namely LNCaP and 22Rv1, and androgen-insensitive, such as DU145 and PC3, prostate cancer cells. MT_1_ and MT_2_ protein levels were analyzed by Western blotting in these cell lines. MT_1_ receptor was detectable in all the cell lines examined (Figure 2A). MT_1_ levels were highest in LNCaP cells, intermediate in DU145, and lowest in 22Rv1 and PC3 cells (Figure 2B). In contrast, MT_2_ protein expression was evident only in PC3 and DU145 cell lines. Androgen receptor (AR) expression was confirmed in androgen-sensitive LNCaP and 22Rv1 cells.

### 2.2. Melatonin Analogue Effects on Prostate Cancer Cell Growth

**Figure 3:**
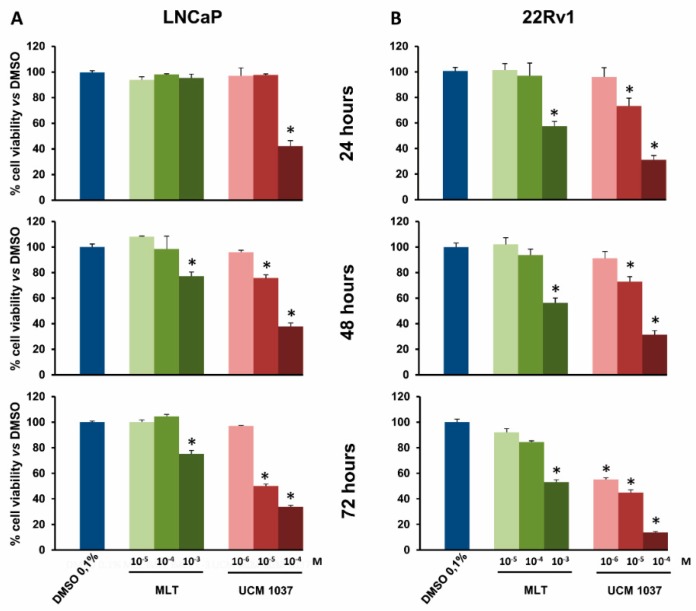
Cell viability of androgen-sensitive prostate cancer cells treated with melatonin and UCM 1037. LNCaP and 22Rv1 androgen-sensitive prostate cancer cells were treated with melatonin (MLT) or with UCM 1037 at the indicated doses expressed in molarity (M), as described in the Materials and Methods. LNCaP (**A**) and 22Rv1 (**B**) cell viability was evaluated after 24, 48, and 72 h. Graphic bars represent the percentage of viable cells in each sample. The results have been normalized to 0.1% DMSO-treated cells and are the means of three independent experiments ± s.d. * *p*-value < 0.001 versus 0.1% DMSO-treated cells.

**Figure 4:**
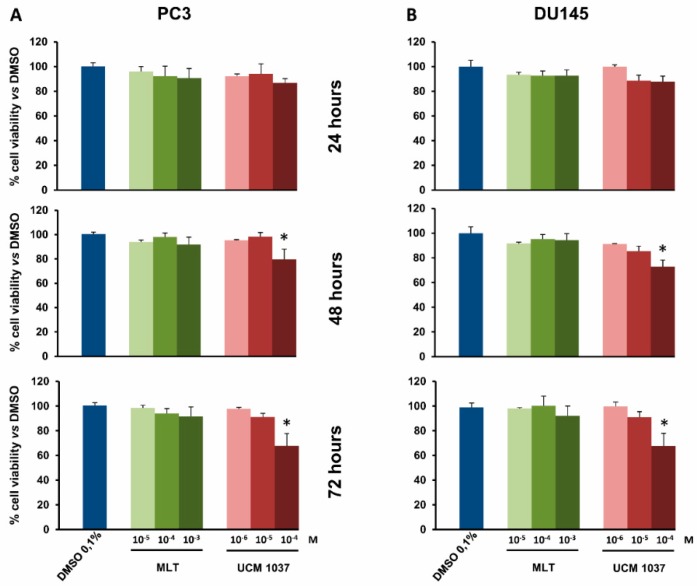
Cell viability of androgen-insensitive prostate cancer cells treated with melatonin and UCM 1037. PC3 and DU145 androgen-insensitive prostate cancer cells were treated with MLT or with UCM 1037 at the indicated doses expressed in molarity (M) as described in Materials and Methods. PC3 (**A**) and DU145 (**B**) cell viability was evaluated after 24, 48, and 72 h. Graphic bars represent the percentage of viable cells in each sample. The results have been normalized to 0.1% DMSO-treated cells and are the means of three independent experiments ± s.d. * *p*-value < 0.001 versus 0.1% DMSO-treated cells.

The antiproliferative effects of UCM 1037 melatonin analogue were evaluated by a cell counting assay that detects cellular metabolic activities. LNCaP, PC3, DU145, and 22Rv1 cells were seeded in 96-well plates, and 24 h later cells were treated with UCM 1037 (10^−6^–10^−4^ M) or with melatonin (10^−5^–10^−3^ M) dissolved in 0.1% dimethyl sulfoxide (DMSO) for 24, 48, and 72 h. As a control, 0.1% DMSO was also administered to cell culture for the same exposure times. The cell viability assay revealed that UCM 1037 had an antiproliferative effect in a dose- and time-dependent manner (Figure 3 and Figure 4). In particular, UCM 1037 10^−4^ M showed the maximum effect in androgen-sensitive cells, reducing LNCaP and 22Rv1 cell number to 38% and 31% after 48 h and to 34% and 14% after 72 h, respectively, compared to DMSO-treated cells (Figure 3A,B).

The melatonin analogue caused a less pronounced decrease in cell proliferation in androgen-insensitive prostate cancer cells, UCM 1037 10^−4^ M reducing cell number to 80% and 73% after 48 h and to 68% and 67% after 72 h in PC3 and DU145 cells, respectively, compared to DMSO-treated cells (Figure 4A,B). Melatonin did not significantly inhibit prostate cancer cell proliferation at the same dose.

### 2.3. Effects of Melatonin Analogue on Cell Cycle Distribution

**Figure 5:**
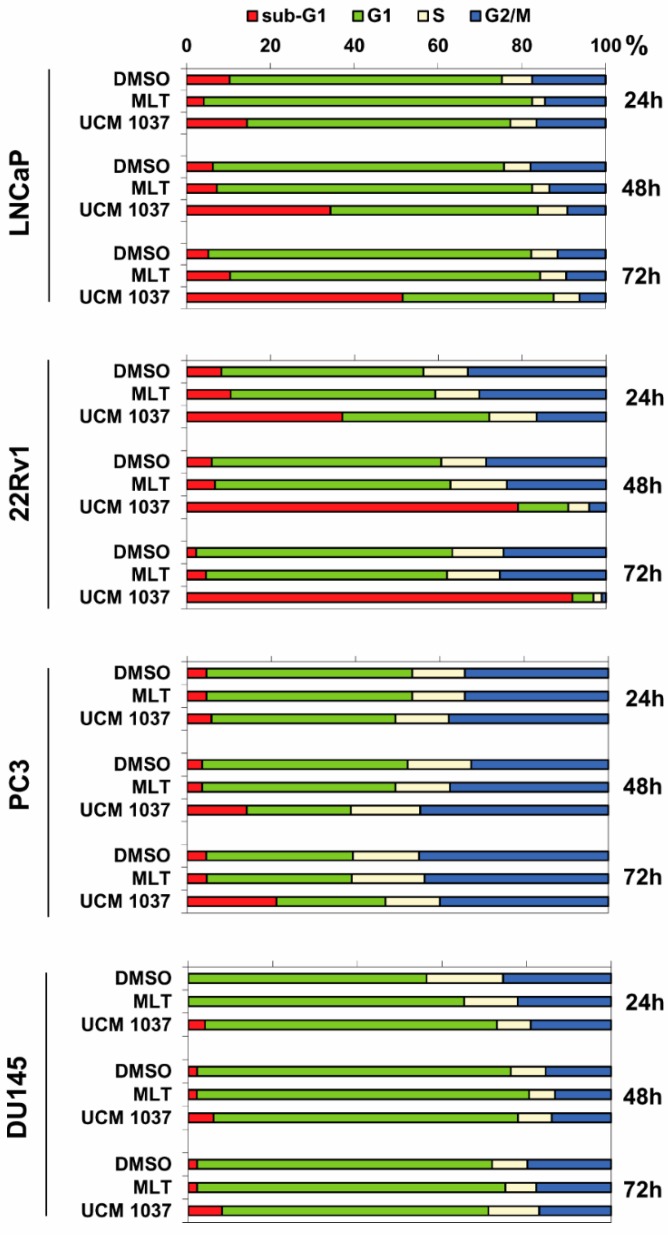
Cell cycle analysis in prostate cancer cells after treatment with melatonin and UCM 1037. LNCaP, 22Rv1, PC3, and DU145 prostate cancer cells were treated with 0.1% DMSO or with MLT (10^−3^ M) or with UCM 1037 (10^−4^ M) for 24, 48, and 72 h, stained with PI, as indicated in the Materials and Methods, and then subjected to flow cytometric analysis. The bar graphs show the percentages of cells in sub-G1 region and G1, S, and G2/M phases. Data are representative of three independent experiments.

To analyze in more detail the antiproliferative effect of UCM 1037 melatonin analogue on prostate cancer cells, we studied the cell cycle distribution of LNCaP, PC3, DU145, and 22Rv1 cells after exposure to 10^−3^ M melatonin or 10^−4^ M UCM 1037 for 24, 48, and 72 h. Cytofluorimetric analysis using propidium iodide (PI) staining revealed a significant and time-dependent accumulation of LNCaP and 22Rv1 cells in the sub-G1 region after UCM 1037 treatment (Figure 5), which is indicative of apoptosis and/or necrosis [18].

UCM 1037 increased the sub-G1 fraction as a function of time in both 22Rv1 (37, 79, and 92% after 24, 48, and 72 h, respectively) and LNCaP (14, 34, and 52% after 24, 48, and 72 h, respectively) cells. In contrast, UCM 1037 only slightly increased the sub-G1 cell population in both PC3 and DU145 prostate cancer cells.

Consistent with the growth inhibitory effect observed in LNCaP, 1 mM melatonin caused a transitory G1 accumulation with a concomitant depletion of S and G2/M phases. Cell accumulation in G1 phase was at a maximum following 24 h of treatment (78% in melatonin-treated versus 65% in control cells), lower by 48 h (75% in melatonin-treated versus 69% in DMSO-treated cells), and after 72 h of incubation, the percentage of cells in G1 phase slightly declined below to control level (74% in melatonin-treated versus 77% in DMSO-treated cells). This transient G1 accumulation induced by melatonin was visible, although to a lesser extent, in DU145 cells.

In spite of growth inhibition induced by 1 mM melatonin in 22Rv1 cells, no differences between untreated and melatonin-treated cells were observed, for any phases of the cell cycle.

As expected, the percentage of cells with hypodiploid DNA content (sub-G1) was similar in absence or in presence of 1 mM melatonin, at all time points analyzed in all cell lines.

### 2.4. Cytotoxicity of Melatonin Analogue on Prostate Cancer Cells

**Figure 6:**
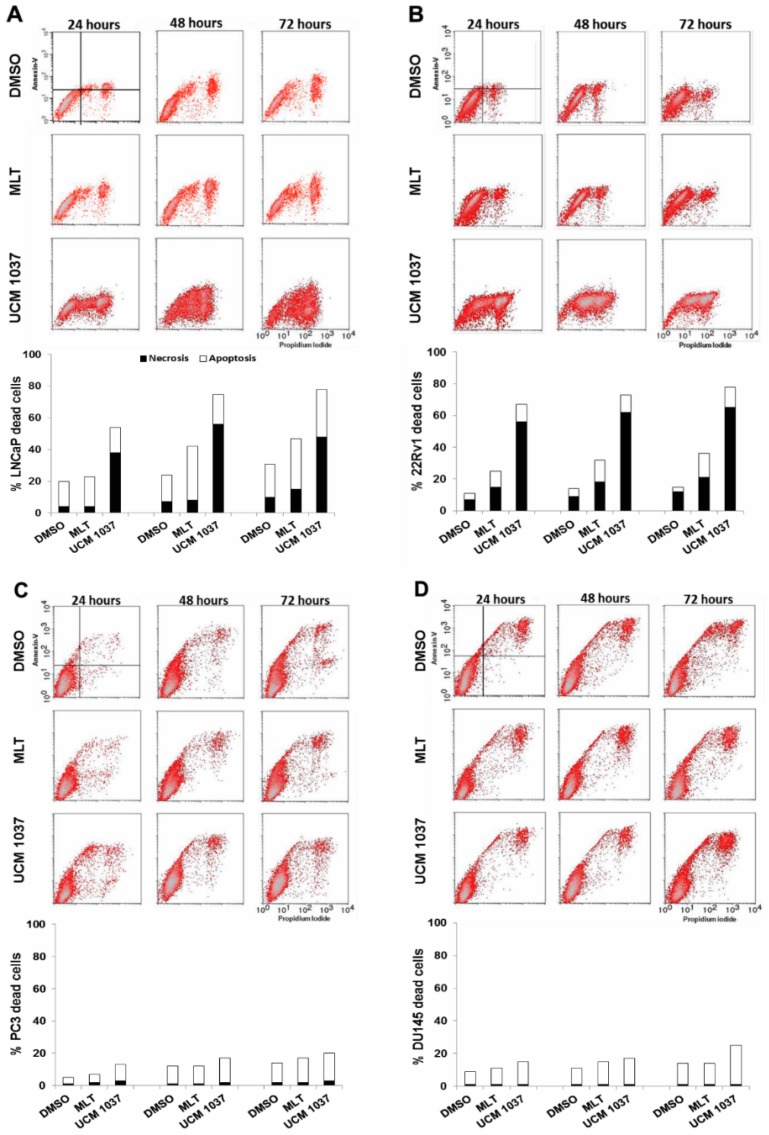
Apoptotic and necrotic cell determinations in prostate cancer cells treated with melatonin and UCM 1037. LNCaP (**A**), 22Rv1 (**B**), PC3 (**C**), and DU145 (**D**) cancer cells were treated with 0.1% DMSO dissolved in culture medium or with melatonin (10^−3^ M) or with UCM 1037 (10^−4^ M) diluted in 0.1% DMSO. After 24, 48, and 72 h cells were simultaneously stained with Alexa Fluor-488-Annexin V and propidium iodide and analyzed by flow-cytometry to determine apoptosis and necrosis as described in the Materials and Methods. One representative experiment out of three performed with similar results is shown. For each panel the cytograms represent viable (Annexin V-negative/PI-negative), early apoptotic (Annexin V-positive/PI-negative), late apoptotic (Annexin V-positive/PI-positive), and necrotic (Annexin V-negative/PI-positive) cells. The bar graphs represent the percentage of early and late apoptotic cells (white bars) and necrotic cells-treated (black bars), as described above.

Apoptosis was then analyzed using Annexin V assay, which enables the detection of apoptotic and necrotic cells by flow cytometry. All the human prostate cancer cell lines were exposed to 10^−3^ M melatonin or 10^−4^ M UCM 1037 for 24 to 72 h and subjected to Annexin V/PI staining. As shown in Figure 6 the distribution of cellular population on the Annexin V/PI cytograms was used to distinguish living cells (Annexin V-negative/PI-negative), early apoptotic cells (Annexin V-positive/PI-negative), late apoptotic cells (Annexin V-positive/PI-positive), and necrotic cells (Annexin V-negative/PI-positive). The percentage of LNCaP and 22Rv1 necrotic cells after UCM 1037 treatment for 24 h was approximately 9.5- and 8-fold that of the control cells, respectively (Figure 6A,B). The number of stained cells increased over time, indicating that 10^−4^ M UCM 1037 had a conspicuous cytotoxic effect on androgen-sensitive prostate cancer cells. At the concentrations of 1 mM melatonin, apoptotic LNCaP and 22Rv1 cells increased by 1.5- and 5-fold, respectively, compared to control cells, after 72 h (Figure 6A,B). Both UCM 1037 and melatonin cytotoxic effects were less evident in androgen-insensitive prostate cancer cells (Figure 6C,D).

### 2.5. Melatonin Analogue Effects on AR and Akt Levels

**Figure 7:**
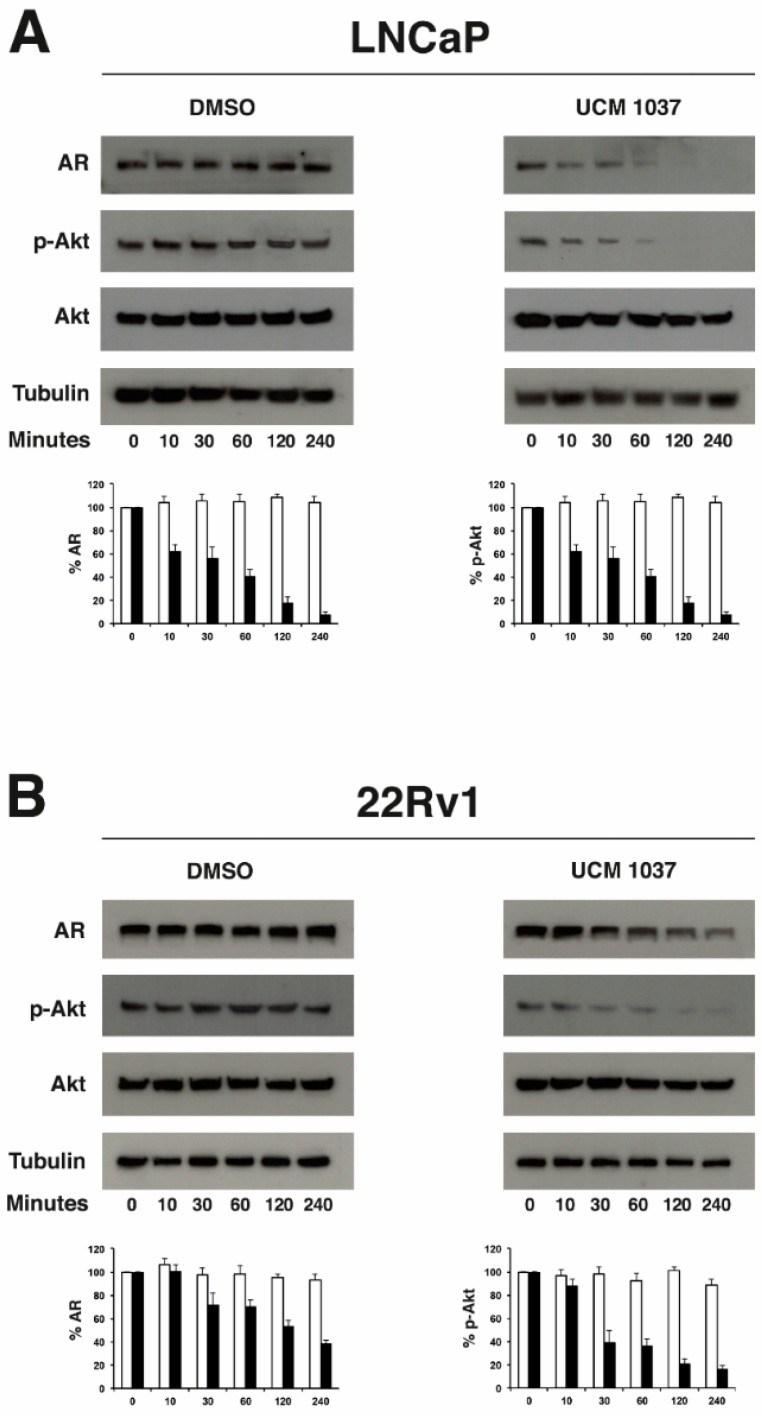
UCM 1037 reduces AR and p-Akt levels in androgen-sensitive prostate cancer cells. LNCaP (**A**) and 22Rv1 (**B**) prostate cancer cells were treated with 0.1% or with UCM 1037 (10^−4^ M) for 0, 10, 30, 60, 120, and 240 min and then subjected to Western blot analysis. Blots are shown for AR, p-Akt, and Akt proteins with their corresponding tubulin controls. One representative blot is shown of three independent experiments. Bar charts show the quantification of AR and p-Akt in DMSO (white bars) and UCM 1037-treated cells (black bars). Densitometric data were normalized versus tubulin and the results are presented as the relative percentage of protein levels at 0 h. Each bar represents the mean ± s.d. of three independent experiments.

To identify the mechanisms underlying UCM 1037-mediated cytotoxicity in androgen-sensitive prostate cancer cells, AR and Akt expression was examined in LNCaP and 22Rv1 cells in a short-term time course (Figure 7). Western blot analysis showed that AR protein levels in UCM 1037 treated cells were significantly decreased in both LNCaP (Figure 7A) and 22Rv1 cells (Figure 7B). Using densitometric analysis, AR protein levels were quantified and normalized versus tubulin levels. The histograms shown in Figure 7 indicate that AR decreases to 7% and 38% in UCM 1037 treated LNCaP and 22Rv1 cells, respectively, after 4 h. No significant changes in AR levels were visible in DMSO-treated cells.

To determine if the melatonin analogue acted on the Akt pathway, Ser473 phosphorylated Akt (p-Akt) levels in LNCaP and 22Rv1 cells were determined. Western blot analysis showed that the melatonin analogue effectively decreased Akt phosphorylation in a time-dependent manner in UCM 1037 treated cells. Densitometric analysis revealed that p-Akt levels declined to 3% and 16% in LNCaP and 22Rv1 cells, respectively, 4 h after UCM 1037 treatment. No changes in total Akt levels were visible in both DMSO and UCM 1037 treated cells.

## 3. Discussion

Many studies suggest that natural bioactive compounds and their synthetic derivatives could be used in combination with traditional chemotherapeutic agents as potential anti-cancer therapies [19,20]. In this study, we employed a melatonin analogue and studied its role in cancer cell proliferation control and the ability to promote cell death in prostate cancer cells.

LNCaP, PC3, DU145, and 22Rv1 cell lines differ in several parameters, including androgen responsiveness and melatonin membrane receptor presence, and reflect a range of prostate cancer progression and androgen sensitivity, which make them excellent models to evaluate the antiproliferative role of melatonin and its derivatives, as well as the possible mechanisms involved.

Melatonin receptor expression has been demonstrated in many cancer types and activation of MT_1_ and MT_2_ receptors has been proposed to induce antiproliferative and pro-differentiating effects, as recently reviewed by Reiter et al. [7]. Here, we demonstrated the presence of G protein-coupled melatonin MT_1_ receptor in all the above mentioned human prostate cancer cell lines, although the membrane receptor levels greatly differ among them (Figure 2). MT_1_ receptor protein expression was previously detected in LNCaP, 22Rv1 and DU145 cells but not in PC3 cells by immunocytochemistry using purified anti-MT_1_ receptor serum [21]. On the contrary, we could establish that PC3 cells express melatonin MT_1_ membrane receptor protein, though to a low level. We also showed that the MT_2_ receptor, which is more restrictively expressed in human cell types and tissues, is present in androgen-insensitive DU145 and PC3 prostate cancer cells, but not in androgen-sensitive LNCaP and 22Rv1 cells.

It has been previously shown that melatonin inhibits the growth of LNCaP and 22Rv1 androgen-sensitive human prostate cancer cells and that the antiproliferative action of melatonin is mediated, at least partially, by MT_1_ activation [14,22]. Melatonin antiproliferative effects on PC3 and DU145 cells are more controversial: the indolic hormone was found to have no effects on the proliferation of androgen-insensitive PC3 and DU145 cells by Siu et al. [21], while others reported inhibitory actions of melatonin on PC3 and DU145 cell proliferation in vitro [23,24,25]. However, no effects of melatonin on the growth of DU145 and PC3 tumors was observed in nude mice [13].

We recently demonstrated that the melatonin receptor ligand UCM 1037 acts as a full agonist in both melatonin receptor subtypes and that this compound is effective in suppressing cancer cell growth of melanoma both in vitro and in vivo [17]. Although UCM 1037 antiproliferative activity could be attributed, at least in part, to the receptor-mediated actions of the melatonin derivative, the hypothesis that it may be modulated by the compound ability to interact with lipophilic compartments of the cells and/or be due to off targets could not be ruled out.

Here we evaluated the effects of the melatonin derivative UCM 1037 on cell proliferation in both androgen-sensitive and androgen-insensitive prostate cancer cells. According to the results of the cell viability assays, UCM 1037 demonstrated substantial dose- and time-dependent antiproliferative effects in androgen-sensitive LNCaP and 22Rv1 cells (Figure 3).

Interestingly, despite the high level of expression of the MT_1_ receptor in DU145 cells (Figure 2), UCM 1037 inhibited androgen-insensitive cell proliferation only at the 10^−4^ M concentration, while melatonin did not elicit any significant effect in androgen-independent prostate cancer cells, thus confirming previously-obtained results [21]. Given that the MT_1_ receptor is considered to be responsible for the transduction of the antiproliferative signal of melatonin [13,22], it is possible that the altered MT_1_ receptor functions and/or interactions in androgen-insensitive PC3 and DU145 cells may account for the failure of melatonin antiproliferative signal transduction.

Overall, we observed that the melatonin derivative exerts antiproliferative effects on MT_1_-positive, MT_2_-negative androgen-sensitive prostate cancer cells. From these data we can infer that UCM 1037 activity on prostate cancer cell proliferation is MT_2_-independent, while MT_1_ expression is not sufficient to elicit this effect. We previously demonstrated that UCM 1037 induced significant inhibition of cell proliferation in NIH3T3-1A and NIH3T3-1B cell clones, expressing MT_1_ and MT_2_, respectively, compared to wild-type NIH3T3 cells [17]. These results indicated that the antiproliferative activity of the melatonin analogue is receptor-dependent and is possibly mediated by both melatonin receptors. However, the UCM 1037 mode of action is stringently cell line-dependent and receptor-independent effects can also be hypothesized.

To further examine whether UCM 1037-induced cell growth inhibition is mediated via alterations in cell cycle progression, we analyzed the effects of the melatonin derivative on cell cycle phase distribution by flow cytometry. The decrease of LNCaP and 22Rv1 cell numbers due to the antiproliferative action of UCM 1037 resulted in induction of cell death, as demonstrated by the high percentage of cells with hypodiploid DNA content (sub-G1) accumulating over time.

The antiproliferative mechanism triggered by UCM 1037 in androgen-sensitive prostate cancer cells is, thus, quite different from melatonin-induced cell proliferation reduction. Indeed, data presented in this study, and previously reported by others, support a melatonin-induced cell growth inhibition mechanism independent of apoptosis induction, but rather reliant on cell accumulation in the G1 phase, in melanoma, breast, and prostate cancer cells [18,25,26,27].

Cytotoxicity induced by UCM 1037 in androgen-sensitive prostate cancer cells was further demonstrated by Annexin V binding combined with PI staining, which serves as a sensitive detection method of early and late stages of apoptosis, as well as necrosis. The percentage of LNCaP and 22Rv1 necrotic cells dramatically increased following UCM 1037 exposure, the apoptotic rate of 22Rv1 cells also augmented compared to DMSO-treated control cells, although to a lesser extent. On the contrary, the UCM 1037 cytotoxic effect was much less evident in androgen-insensitive prostate cancer cells. Taken together, the flow cytometry data presented herein suggest that UCM 1037 is highly cytotoxic to androgen-sensitive prostate cancer cells, although we did not observe a substantial increase in the apoptotic cell fraction, in contrast to what we observed in melanoma cells [17].

These findings prompted us to further investigate the effects of UCM 1037 in the signaling pathways involved in antiproliferative and cytotoxic actions of the melatonin derivative.

Recently, a functional link between the androgen receptor and melatonin signal transduction pathways has been envisaged. In particular, Zisapel’s group demonstrated that melatonin induces nuclear exclusion of the AR via activation of protein kinase C in androgen-insensitive prostate carcinoma PC3 cells stably transfected with a wild-type AR-expressing vector [28,29]. Moreover, knockdown of the expression of AR in LNCaP and 22Rv1 cells resulted in abrogation of melatonin receptor-mediated cell proliferation inhibition, indicating that the antiproliferative signaling pathway activated by melatonin in human prostate cancer cells is AR-dependent [30]. These data indicate the presence of a cross-talk between MT_1_ receptor and AR signaling in prostate cancer cells. However, melatonin actions are not limited to being merely anti-androgenic, as recently demonstrated by comparative genome microarray, melatonin-treated and androgen-deprived cells show many differentially expressed genes [31].

For these reasons we evaluated AR expression in UCM 1037-treated LNCaP and 22Rv1 cells and what we found was a remarkable down regulation of AR protein levels, which has never been observed before in melatonin-treated prostate cancer cells. These findings emphasize again that UCM 1037 and melatonin molecules elicit different responses on downstream signaling pathways.

Due to the lipophilic nature of UCM 1037 [17], receptor-independent effects could also be envisaged, with multiple signaling leading to modulation of different cascades. Several studies indicated that the PI3K/Akt cascade is associated with melatonin-mediated antiproliferative actions, although both stimulation [32,33] and inhibition [34,35,36,37] of phosphorylated Akt have been observed using different cell types.

We previously highlighted that the melatonin analogue UCM 1037, as well, can trigger and/or inhibit Akt phosphorylation differently in the cell lines examined: p-Akt increased in melanoma DX-3 cells, remained unaffected in melanoma WM-115 cells and dramatically decreased in breast cancer cell lines [17]. Here, we observed that UCM 1037 strongly inhibited Akt phosphorylation in androgen-sensitive prostate cancer.

Interestingly, a cross-talk between AR and Akt has been recently demonstrated in prostate cancer, whereby inhibition of Akt in cells expressing high levels of p-Akt resulted in decreased AR protein levels, overexpression of Akt resulted in increased levels of AR protein, while inhibition of low levels of endogenous Akt kinase activity did not affect AR protein levels [38].

According to the data presented here, p-Akt decrease paralleled AR reduction in LNCaP and 22Rv1 cells treated with UCM 1037, regardless of basal p-Akt levels. In fact, Akt phosphorylation is substantially up-regulated in PTEN-deficient LNCaP cells [39].

In view of the above, two not mutually exclusive scenarios can be considered to tentatively explain the molecular mechanisms underlying UCM 1037 antiproliferative and cytotoxic effects in androgen-sensitive prostate cancer cell lines: UCM 1037 may directly down-regulate AR and p-Akt levels, or AR down-regulation may be triggered by inhibition of Akt activation. However, the latter hypothesis alone would probably not suffice the almost simultaneous decrease in AR and p-Akt levels.

Although studies presented in this report did not examine MT_1_’s role in mediating UCM 1037 effects, no correlation could be found between MT_1_ levels and the melatonin derivative antiproliferative and cytotoxic actions.

Mechanistically, we can conclude that UCM 1037 melatonin derivative inhibits androgen-sensitive prostate cancer cells growth, exerts a cytotoxic effect, down-regulates AR levels and Akt activation, possibly via a melatonin receptor-independent mechanism.

Together with the previous findings reported by this and other laboratories, data presented here on melatonin analogue anticancer effects may have significant implications on future clinical exploitation of these molecules in prostate cancer therapeutics.

## 4. Materials and Methods

### 4.1. Cells Culture and Reagents

The human prostate cancer cell line LNCaP [40], obtained from ICLC (Genova, Italy), was maintained in RPMI 1640 medium (Euroclone, Milan, Italy) supplemented with 20% FCS (Fetalclone I, Hyclone, Logan, UT, USA), 2 mM l-glutamine, 100 U/100 μg/mL penicillin/streptomycin (Sigma-Aldrich, Milan, Italy), 10 mM HEPES (Sigma-Aldrich). PC3 [41], and DU145 [42], obtained from Dr. N. Zaffaroni (Fondazione IRCCS Istituto Nazionale Tumori, Milan, Italy), and 22Rv1 [43], obtained from ATCC (Manassas, VA, USA) human prostate cell lines were maintained in RPMI 1640 medium supplemented with 10% FCS, 2 mM l-glutamine, 100 U/100 μg/mL penicillin/streptomycin solution. All cell lines were maintained at 37 °C and 5% CO_2_. Melatonin was purchased from Sigma-Aldrich and UCM 1037 was kindly provided by Dr. G. Spadoni (University of Urbino, Italy).

### 4.2. Cellular Proliferation and Viability Assay

LNCaP, 22Rv1 (4 × 10^3^/well), PC3 and DU145 (2 × 10^3^/well) cells were seeded in 96-well plates and incubated at 37 °C with a 5% CO_2_ humidified atmosphere. 24 h later, cells were treated with 0.1% DMSO dissolved in culture medium or with different doses of melatonin and UCM 1037 diluted in 0.1% DMSO and cultured for 24, 48, and 72 h.

The cell viability and proliferation were assayed using the Cell Counting Kit-8 (Dojindo Laboratories, Japan), according to the manufacturer’s protocol. Briefly, cells were incubated for four hours with freshly prepared WST-8 solution (10 µL/well) and then the number of viable cells was assessed by measuring the absorbance at 450 nm, using a Wallac Victor 1420 Multilabel Counter (Perkin Elmer, MA, USA). The experiments were performed in quadruplicate and repeated three times.

### 4.3. Cell Cycle Analysis by Flow Cytometry

LNCaP, 22Rv1 (5 × 10^5^), PC3 and DU145 (3 × 10^5^) cells were seeded in 60-mm plates and 24 h later they were treated with 0.1% DMSO dissolved in culture medium or with melatonin (10^−3^ M) or with UCM 1037 (10^−4^ M) diluted in 0.1% DMSO. After 24, 48, and 72 h cells were collected, washed, and stained for 30 min at 37 °C with 1 mL of DNA-staining solution in 0.1% Nonidet P-40 (Sigma-Aldrich, Milan, Italy), 0.5 mg/mL RNAse (type IIIA, Sigma-Aldrich), and 25 µg/mL propidium iodide (Sigma-Aldrich) [44]. The cellular DNA content was analyzed by FACScalibur (Becton Dickinson Immunocytometry Systems, San Jose, CA, USA) using a Cell Quest (Becton Dickinson) software system for histograms of propidium iodide fluorescence intensity vs. cell frequency.

### 4.4. Detection of Apoptosis and Necrosis by Annexin-V/Propidium Iodide Assay

LNCaP, 22Rv1 (5 × 10^5^), PC3, and DU145 (3 × 10^5^) cells were seeded in 60-mm plates and incubated at 37 °C with a 5% CO_2_ humidified atmosphere. 24 h later, cells were treated with 0.1% DMSO dissolved in culture medium or with melatonin (10^−3^ M) or with UCM 1037 (10^−4^ M) diluted in 0.1% DMSO. After 24, 48, and 72 h cells were simultaneously stained with Alexa Fluor 488-conjugated Annexin-V and PI, using the Vybrant Apoptosis Assay kit #2 (Molecular Probes, Eugene, OR, USA), according to the manufacturer’s instructions. Samples were analyzed by FACScalibur flow cytometer (Becton Dickinson). In each analysis 10,000 events were recorded and the percentage of apoptotic cells estimated by means of the CellQuest Pro software (Becton Dickinson). The simultaneous staining of cells with Annexin-V and PI allowed the resolution of viable cells (Annexin V-negative/PI-negative), early apoptotic cells (Annexin V-positive/PI-negative), necrotic cells (Annexin V-negative/PI-positive), and late apoptotic cells (Annexin V-positive/PI-positive).

### 4.5. Western Blot Analysis

LNCaP and 22Rv1 cells were plated onto 60-mm dishes at a density of 5 × 10^5^ cells/mL and incubated at 37 °C with 5% CO_2_ humidified atmosphere. Twenty-four hours later, cells were treated with 0.1% DMSO dissolved in culture medium or with 10^−4^ M UCM 1037 diluted in 0.1% DMSO and cultured for 10, 30, 60, 120, and 240 min, then harvested. Cells were collected by centrifugation at 1000 rpm for 10 min, washed with cold PBS, resuspended in lysis buffer (50 mM Hepes-NaOH, pH 7.5, 150 mM NaCl, 15 mM MgCl_2_, 1 mM EGTA-NaOH pH 7.5, 1% Triton X-100, 1 mM sodium orthovanadate, 1 mM phenylmethylsulfonyl fluoride, and 10 μg/mL of leupeptin, aprotinin, antipain, and chymostatin) and incubated on ice for 45 min. The lysates were centrifuged at 13,000 rpm for 10 min at 4 °C. Equivalent amounts of proteins were analyzed by SDS–polyacrylamide gel electrophoresis. After electrophoretic separation, the proteins were transferred onto nitrocellulose membrane (GE Healthcare, Pittsburg, PA, USA). After 1 h of incubation in blocking solution (5% milk in PBS/0,1% Tween), filters were incubated with the appropriate antibodies: MT_1_, MT_2_ (Biorbyt, San Francisco, CA, USA), Ser473 phosphorylated Akt (p-Akt), Akt, Androgen Receptor (Cell Signaling Technology, Danvers, MA, USA) and β-Tubulin (Sigma-Aldrich). Proteins were visualized with peroxidase-coupled secondary antibody (GE Healthcare), using enhanced chemiluminescence (ECL) for detection (GE Healthcare). Densitometry was performed on scanned immunoblot images using the NIH ImageJ software (NIH, Bethesda, MA, USA).

### 4.6. Statistical Analysis

Data are the mean ± standard deviation of at least three independent experiments and were evaluated by Student’s *t*-test. Values of *p* < 0.001 were considered statistically significant.
